# Medical News and Miscellany

**Published:** 1891-08

**Authors:** 


### Medical News and Miscellany.

---

Dr. J. L. Lankford has removed from Atoka, I. T., to San Antonio on account of impaired health of self and wife.

---

Dr. H. A. West, Secretary Texas State Medical Association and Professor of Practice in the Texas Medical College, is in New York, and will return about September 20th.

**Dr. T. O. Summers**, well-known to the American profession as a brilliant writer and journalist, has accepted the appointment of resident physician at the Fountain House, Waukesha.

---

**All Communications** and Exchanges for the Texas State Medical Association should be addressed to Dr. H. A. West, Galveston, Secretary, *vice* the editor of this JOURNAL resigned.

---

**Our esteemed contemporary**, the *N. O. Medical and Surgical Journal*, under the management of Dr. McShane, has donned a new dress and comes to us greatly improved in appearance and in contents.

---

**Dr. R. M Swearingen**, State Health Officer, and family, are spending some time at Corpus Christi for the Doctor's health. In the early part of August he had a severe attack of pleuritis with effusion, from the effects of which he is still suffering.

---

**Under the** discriminating and æsthetic management of Dr. Wm. Porter, of the St. Louis College of Physicians and Surgeons, the *Clinique* is one of the brightest, best and most readable of the exchanges that come to our table. It is a model journal.

---

**Let** students not forget our offer to pay for first course of lecturers at Medical Department University of Texas in return for one hundred and forty subscribers to the JOURNAL by November 1. Send names and money as secured, keeping one half ($1) of each subscription, so that if you do not get one hundred and forty you get paid for all you do get.

---

**A busy man**—H. M. Whelpley, M. D., Ph. S. F. R. M. S., has been a lecturer in the Missouri Medical College for the past five years. He is now Professor of Physiology and Histology, Director of the Histological Laboratory, and Secretary of the Faculty. The Doctor is also editor of the *Meyer Brothers Druggist*, and Professor of Microscopy in the St. Louis College of Pharmacy.

---

**Tri-State Medical Association.**—The third annual meeting of the Tri-State Medical Association will convene in Turner Hall, Chattanooga, Tenn., Tuesday, October 27th, 1891, and continue in session three days. Indications are that it will be one of the largest medical meetings ever held in the South. Representative physicians from all sections will be present.

All who desire to read papers, should send title to the secretary of the association before September 1st. In due time a circular will be issued giving a complete list of all papers, and names of exhibitors who apply for space before October 1st.

W. L. GAHAGAN, Sec'y of Executive Com.,
P. O. Box 542. Chattanooga, Tenn.

---

Dr. D. R. Wallace the founder and long time Superintendent of the State Lunatic Asylum at Terrell, has a card to the profession in this issue. His practice will be confined to the treatment of and consultation on the diseases with which his extensive reading and long experience render him familiar,—diseases of the mind and nervous system. He solicits correspondence with physicians needing counsel in such cases. No man in Texas has larger claims to be regarded as authority on insanity and kindred diseases than he; and when it is remembered that there is no private institution in Texas for the care of insane persons of the better class, the state asylum being always full, and preference in case of vacant room, being given by law to the indigent insane, a valuable resource here presents, of which the intelligent physician will not be slow to avail himself. Read the card.

---

The *American Journal of Surgery*, Vol. 1, No. 2, has been received. It is edited and published by Dr. E. Lamphear, Kansas City, and is the latest candidate, we believe, for favor. The Doctor is to be congratulated upon its get-up, and upon successfully conducting two such creditable publications as this and the *Medical Index*. He is a man of ability, and can manage more irons in the fire without burning, than any one we know. In addition to his journals, he conducts a large Infirmary, where recently he has successfully performed several laparotomies. He is also a member of the Faculty of the Kansas City Medical College and has to lecture several times a week. We have never met the Doctor—and in our imagination we fancy we see a big man with as many eyes as Mr. Argus was said to have had, and as many arms as Mr. Briarius—and a very Hercules in strength.

---

"Wiring the Vertebræ as a means of Immobilization in Fracture and in Potts' Disease." At the Waco meeting of the Texas

State Medical Association in April 1891, Prof. B. E. Hadra, M. D., of Galveston, Prof. of Surgery in the Texas Medical College, read a paper on this subject, which is published in the yearly volume of Transactions just issued. A few reprints were issued, and the paper has attracted much attention and excited wide interest. The proposition to wire the bones of the vertebra is something new; and as Dr. T. D. Wooten, President Board of Regents Texas State University said, in discussing the paper, "It furnishes a resource in a certain class of cases where surgical assistance is sometimes very urgently needed, and when the means at our disposal heretofore, were inadequate. The paper is an able and practical one, and the means suggested for the treatment of certain diseases and injuries of the spine are worthy of further trial and experiment.'' And the paper, we will add, is worthy of a wider circulation than is afforded by the limited edition of the Transactions, hence to oblige many who have asked for it, as well as to give it greater currency, we will reproduce it in the September nunber of this journal—a double edition.

---

**Dr. Holmes' Private Infirmary at Rome, Ga.**— In this issue appears an excellent cut of this beautiful building and its picturesque surroundings, with a card to the profession. Dr. Holmes is President of the Georgia State Medical Association, and is well and favorably known to the profession throughout the United States. As a gynecologist he deservedly ranks high and, especially in the South, he is regarded as an authority, ranking second only to the immortal Sims. The climate of Rome is propitious, the air pure and healthful, the scenery attractive; and thus surrounded, and in such skillful hands the Infirmary offers rare inducements to physicians to send patients there who require gynecological treatment. The building is large and airy, and has been constructed under Dr. Holmes' personal supervision, with every attention to hygiene and the personal comfort of those entrusted to his care. The institution is first-class in every particular, and the high character of the manager as an operator and physician, is sufficient guarantee that nothing will be left undone to insure success in the treatment of the surgical diseases of women.

---

**Kind Words Cheer the Hearts of the Weary.**–The following high testimonial to the JOURNAL comes unsolicited from one of the oldest and most esteemed practitioners of West Texas,—a man who has spent forty years in the cause of humanity. Such expressions of appreciation are most cheering; they put a new point to the editor's pencil, and strengthen his arm; they make him feel that he has not worked in vain: "My sympathies are, and have ever been with the Association, and with your JOURNAL in your praiseworthy efforts to elevate the standard of medicine, and enforce observance of the letter and the spirit of the code. Allow me to congratulate you on the continued improvement of the JOURNAL. It is the "TEXAS JOURNAL" parexcellence and sui-generis, has no superior and withal is "up to time." It is an honor to the Profession in the State, to the city of Austin, and to its editor. Such is my candid opinion and I give it unsolicited." C. S. REEVES, M. D.

---

Tales of a Country Doctor.—A handsome new edition of Dr. King's rare book has just been issued by Dr. Hummel of Philadelphia, in embossed cloth cover with gilt letters, illustrated profusely with new cuts. It is the most rapid selling book since the days of "Uncle Tom's Cabin." We have arranged with the publishers to furnish it to subscribers of this journal at 50c. per copy; that is, to new subscribers, or to those who promptly renew their subscriptions, remitting for Vol. 7 in advance. Enclose us $2.50 and the JOURNAL will be sent you one year, and a copy of the book post paid will be mailed you on receipt of order. We also furnish Lanphear's *Kansas City Medical Index* free to new subscribers who remit $2 in advance for Vol. 7.

In this connection we beg to remind our friends that in the majority of cases, their subscription expires with the June No. (The volume begins with July each year,) but owing to hard times we have not sent out bills for Vol. 7. Nevertheless, we will be very glad to have our subscribers remit if convenient.

---

Transactions Texas State Medical Association 1891. (Waco meeting). The *Journal* is in receipt of a copy of the Transactions of the last meeting of the State Medical Association, gotten out under the new *regime*, edited and published (in Galveston) by the new Secretary, Dr. H. A. West, chairman of the publishing committee; and it affords us a real pleasure to testify to the excellence of the work. The volume is most creditable to the committee and to the Association, notwithstanding Dr.

West is, as he says, "new to this business." It is also issued much earlier than usual. The volume is the smallest issued since 1884; it contains 278 pages, is printed on clean nice paper, and is a model of typographic neatness. It is issued from the press of Clark & Courts of Galveston, and they have reason to be proud of the job. The *Journal* acknowledges the courtesy of an extra-bound copy in pressed morocco and gilt letters. The edition is handsomely bound in brown cloth, and colored edges.

Dr. West has reversed the usual order, and the Constitution and By-laws, which, the latter says, "shall be attached to each volume," appear in front, along with the roll of members ; the body of the volume, the proceedings and papers and addresses etc. coming after. We fail to appreciate the reasons assigned by the Secretary for the change,—but it is a change.

Of the contents we will have something to say later. The papers are about up to the usual average ; one or two being of unusual interest and importance, will be reproduce in this journal.

---

Vol. 7.—How "tempus" does "fugit" ! Six years ago the JOURNAL was launched on the "uncertain sea," and results were watched with much anxiety. In a remarkably short time its position in the front rank was attained, and universally conceded. It begins its seventh "round" under most favorable auspices, —enlarged and otherwise improved ; comes out in a new, and brighter, and still redder dress, which shows that the fires of enthusiasm in the cause of *reform*, organization, and the advancement of Rational Medicine, have not waned,—but burn as brilliantly as in the beginning. Nor has the interest aroused in the profession by its advent, lessened. The better class of the Texas profession gave it at once a cordial recognition as the exponent of legitimate medicine, and have been steadfast in their support. To its many friends, old and new, the JOURNAL makes its nicest bow ; and the management tenders its most grateful and graceful acknowledgment. It has met some ground swells, it is true,—encountered some rough weather,—*raised* several small sized tempests,—has been shot at,—ambushed and bushwhacked ; but right gallantly has it ridden out the storms, and goes forward to-day—as gallantly and undaunted as when it was first "launched," and flung its red banner to the breeze. "Success" is inscribed on that banner, and "Organization",—"Legitimate Medicine" and "Reform" are its keynotes;—to wage unceasing war on quackery—whether in or out the pale of organization—and to insist on an observance of the spirit and letter of the code, will be its steadfast purpose.

Send along your orders; begin now; and as times are hard we will not ask you for pay till you sell your yearlings or collect your hard-earned doctor's bills.

---

**Higher Education.**—At a recent meeting of the Faculty of the Marion-Sims College of Medicine, the Dean, Dr. Young H. Bond, introduced the following resolutions, which were unanimously adopted:

WHEREAS, the position taken by this College upon the two questions of Medical Legislation and Medical Education has been intentionally confounded; and,

WHEREAS, notwithstanding the fact that, at the last meeting of the Missouri State Medical Association, the report on Medical Education offered by Dr. McAlister, and having as its central idea a three years graded course of lecture, was, on motion of your Dean, with the aid of the votes of all the members of this Faculty then present, adopted, it has been sought to have it appear that this College is not favorable to higher Medical Education; therefore, to the end that our position upon the question of Medical Education be clearly understood, be it

*Resolved*, that after the session of '91–'92 the Marion-Sims College of Medicine will exact as a condition to graduation in Medicine of all its students, who may not have previously matriculated, attendance upon a graded course of lectures extending over three years; and be it further

*Resolved*, that our position upon the question of Medical Education does not in the least abate or compromise our objection to what we regard as the attempted enactment of unjust, inefficient and class Medical Legislation, and that this Faculty favors an Examining Board as the fair and rational solution of the problem of Medical Legislation and Medical Education as well.

---

**The Election of Professors** for the new medical college (Medical Department, University of Texas), is at this moment, 10 a. m., August 27, in progress in Galveston. The JOURNAL has held a form open till this late hour in the hope of being able to give its readers the result,—to be the first journal to make the announcement; but as there has been a tie, between Dr. Seth M.

Morris, of Austin, and Dr. Brackett (recommended by Prof. Mallett, of University of Virginia), for the chair of chemistry, as announced by a telegram to the JOURNAL last night, we are obliged to go to press without a report. The Board of Regents met in Galveston at 10 a. m., on the 25th, but as there were a great many applications to examine it has not been possible for them to get through; but to-day will decide the matter. It will be remembered that Drs. Paine and West were elected respectively to the chairs of Practice, and Obstetrics, some weeks ago; six chairs remaining to be filled at this session. We learn from private sources that Dr. Finney, of the Johns-Hopkins University, has been elected Professor of Surgery. As to other chairs—with the exception of Chemistry and Anatomy,—it were mere speculation to attempt to name the coming professors; but, as there was a tie between the foremost candidates for Chemistry, it is believed that neither will be elected, and that a dark horse —perhaps Lee, of Arkansas,—will come in; and the general impression is, with regard to the chair of Anatomy—that the brilliant and popular Geo. H. Lee will be elected to that chair,—he having so crèditably filled the position in the late Texas Medical College.

---

Do not forget that the next regular quarterly meeting of the enterprising *Austin District Medical Association* will be held on September 24th, ult. An attractive program has been arranged and will be published in a few days. The discussions will be very interesting. Do not fail to attend. Nearly one hundred working members.

